# Supplementary material for: Disentangling age-dependent DNA methylation: deterministic, stochastic, and nonlinear
Source: Sci Rep. 2021 Apr 28;11:9201. doi: 10.1038/s41598-021-88504-0 (PMC8080842; doi:10.1038/s41598-021-88504-0)
Supplement: Supplementary file 1 — Supplementary Information. [file 41598_2021_88504_MOESM1_ESM.pdf]

Disentangling age-dependent DNA methylation: deterministic, stochastic, and nonlinear

O. Vershinina[1,*], M.G. Bacalini[2], A. Zaikin[1,3,4], C. Franceschi[1,2], and M. Ivanchenko[1]

[1]Department of Applied Mathematics, Mathematics of Future Technologies Center, Laboratory of Systems Medicine of Healthy Aging, Lobachevsky University, Nizhny Novgorod, 603950, Russia

[2]University of Bologna and IRCCS Istituto delle Scienze Neurologiche di Bologna (ISNB), Bologna, 40139, Italy

[3]Department of Mathematics and Institute for Women's Health, University College London, London, WC1H 0AY, UK

[4]Centre for Analysis of Complex Systems, Sechenov First Moscow State Medical University (Sechenov University), Moscow, 119992, Russia

[*] olya.vershinina@itmm.unn.ru

SUPPLEMENTARY MATERIALS

Supplementary Figures

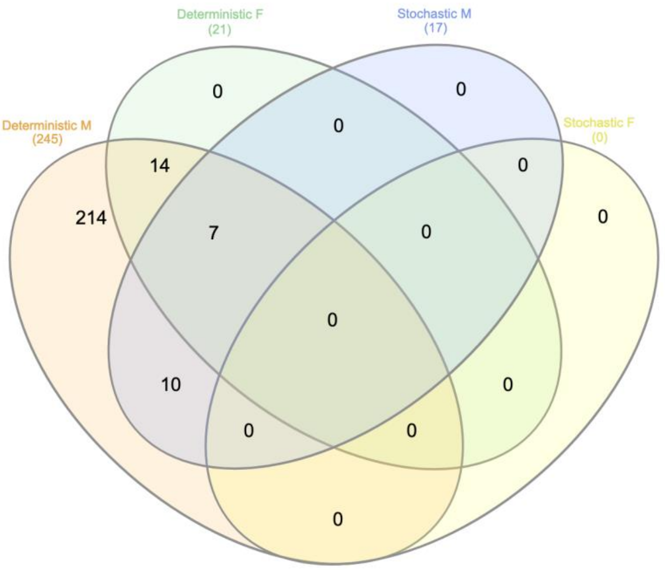

Supplementary Figure 1. Venn Diagram of the significantly enriched GO in deterministic and stochastic aVMPs identified in males (M) and females (F).

Supplementary Files

Supplementary File 1. Lists of age-associated variably methylated positions, aVMPs, (column *"name_cpg"*) computed for beta values. These lists were compared with similar lists obtained for residuals of methylation values (Supplementary File 3, intersecting probes are marked with "X" in the *"isIntersect"* column).

Supplementary File 2. Gene Ontology enrichment of aVMPs lists (obtained for beta values).

Supplementary File 3. Lists of age-associated variably methylated positions, aVMPs, (column *"name_cpg"*) computed for residuals of methylation values. These lists were compared with similar lists obtained for beta values (Supplementary File 1, intersecting probes are marked with "X" in the *"isIntersect"* column).

Supplementary File 4. Lists of age-associated variably methylated positions, aVMPs, computed for beta values (column *"name_cpg"*) divided into deterministic and stochastic probes (column *"type"*). Subsets of deterministic and stochastic CpGs were compared with similar subsets obtained for residuals of methylation values (Supplementary File 6, common deterministic CpGs for beta values and residuals are marked with "X" and common stochastic CpGs are marked with "XX" in the column *"isIntersect"*).

Supplementary File 5. Gene Ontology enrichment of deterministic and stochastic lists of probes (obtained for beta values).

Supplementary File 6. Lists of age-associated variably methylated positions, aVMPs, computed for residuals of methylation values (column *"name_cpg"*) divided into deterministic and stochastic probes (column *"type"*). Subsets of deterministic and stochastic CpGs were compared with similar subsets obtained for beta values (Supplementary File 4, common deterministic CpGs for beta values and residuals are marked with "X" and common stochastic CpGs are marked with "XX" in the column *"isIntersect"*).

Supplementary File 7. Lists of age-associated differentially methylated positions, aDMPs, (column *"name_cpg"*) computed for beta values. These lists were compared with similar lists obtained for residuals of methylation values (Supplementary File 9, intersecting probes are marked with "X" in the column *"isIntersect"*).

Supplementary File 8. Lists of age-associated differentially and variably methylated positions, aDaVMPs, (column *"name_cpg"*) computed for beta values. These lists were compared with similar lists obtained for residuals of methylation values (Supplementary File 10, intersecting probes are marked with "X" in the column *"isIntersect"*).

Supplementary File 9. Lists of age-associated differentially methylated positions, aDMPs, (column *"name_cpg"*) computed for residuals of methylation values. These lists were compared with similar lists obtained for beta values (Supplementary File 7, intersecting probes are marked with "X" in the column *"isIntersect"*).

Supplementary File 10. Lists of age-associated differentially and variably methylated positions, aDaVMPs, (column *"name_cpg"*) computed for residuals of methylation values. These lists were compared with similar lists obtained for beta values (Supplementary File 8, intersecting probes are marked with "X" in the column *"isIntersect"*).

Supplementary File 11. Lists of age-associated differentially and variably methylated positions, aDaVMPs, computed for beta values (column *"name_cpg"*) divided into three functional groups: "CV2" ($\sigma^2 \sim \beta^2$), "Fano" ($\sigma^2 \sim \beta$) and "NA" (in column *"type"*). Subsets of "CV2" and "Fano" CpGs were compared with a similar subsets obtained for residuals of methylation values (Supplementary File 12, common "CV2" CpGs for beta values and residuals are marked with "X" and common "Fano" CpGs are marked with "XX" in the column *"isIntersect"*).

Supplementary File 12. Lists of age-associated differentially and variably methylated positions, aDaVMPs, computed for residuals of methylation values (column *"name_cpg"*) divided into three functional groups: "CV2" ($\sigma^2 \sim \beta^2$), "Fano" ($\sigma^2 \sim \beta$) and "NA" (in column *"type"*). Subsets of "CV2" and "Fano" CpGs were compared with a similar subsets obtained for beta values (Supplementary File 11, common "CV2" CpGs for beta values and residuals are marked with "X" and common "Fano" CpGs are marked with "XX" in the column *"isIntersect"*).

Supplementary File 13. Lists of CpGs with nonlinear (power law) methylation change (column *"name_cpg"*) computed for beta values. These lists were compared with similar lists obtained for residuals of methylation values (Supplementary File 14, intersecting probes are marked with "X" in the column *"isIntersect"*).

Supplementary File 14. Lists of CpGs with nonlinear (power law) methylation change (column *"name_cpg"*) computed for residuals of methylation values. These lists were compared with similar lists obtained for beta values (Supplementary File 13, intersecting probes are marked with "X" in the column *"isIntersect"*).
